# Effectiveness of biologic therapies in achieving treatment targets in inflammatory bowel disease; real-world data from the Middle East (ENROLL study)

**DOI:** 10.3389/fphar.2024.1388043

**Published:** 2024-10-16

**Authors:** Mohammad Shehab, Ahmad Alfadhli, Israa Abdullah, Wrood Alostad, Alaa Marei, Fatema Alrashed

**Affiliations:** ^1^ Department of Internal Medicine, Mubarak Al-Kabeer University Hospital, As Sālimīyah, Kuwait; ^2^ Department of Translational Research, Dasman Institute, Kuwait City, Kuwait; ^3^ Department of Pharmacy Practice, College of Pharmacy, Kuwait University, Kuwait City, Kuwait

**Keywords:** surgery, hospitalization, steroids, endoscopic, remission, biologics, inflammatory bowel disease

## Abstract

**Background:**

Real-world data assessing the effectiveness of biologics in patients with inflammatory bowel disease (IBD) in the Middle East are not well-established. In our study, we evaluated the effectiveness of biologic therapies in achieving clinical and endoscopic outcomes in biologic-naïve patients with IBD.

**Design:**

A retrospective chart review was conducted at two tertiary care gastroenterology centers using electronic medical records of patients with moderate-to-severe IBD. The study period was from October 2017 to October 2023. Patients who were on infliximab, adalimumab, ustekinumab, or vedolizumab for 12 months were included in the analysis. The primary outcomes were the percentage of IBD-related hospitalizations or surgeries, achieving steroid-free remission, and endoscopic remission.

**Results:**

A total of 422 patients were included in the study, of whom 264 (62.5%) patients had Crohn’s disease (CD) and 158 (39%) had ulcerative colitis (UC). In patients with CD, endoscopic remission was attained in 51 (52%) of the patients on adalimumab, 38 (53%) of the patients on infliximab, 34 (56%) of the patients on ustekinumab, and 16 (51%) of the patients on vedolizumab. In patients with UC, endoscopic remission was attained in 40 (56%) of the patients on infliximab, 26 (61%) of the patients on adalimumab, 8 (55%) of the patients on ustekinumab, and 11 (53%) of the patients on vedolizumab.

**Conclusion:**

adalimumab, infliximab, ustekinumab, and vedolizumab were all effective in achieving clinical and endoscopic clinical outcomes in IBD in both UC and CD. The findings of this study suggest that the efficacy of biologics in a Middle Eastern population is similar to that in a Western population.

## Introduction

Inflammatory bowel disease (IBD), including Crohn’s disease (CD) and ulcerative colitis (UC), are immune-mediated disorders characterized by chronic inflammation of the gastrointestinal (GI) tract. Over recent decades, the treatment of IBD has changed considerably, culminating in the use of biologic therapies in the late 1990s (Alatab et al., 2020). With the increasing availability of biosimilars and the resulting reduction in cost, it is estimated that the use of biologic therapy in IBD is likely to increase (Anisdahl et al., 2021).

The goals of treatment of IBD are inducing and maintaining remission. Treatment of CD and UC, the two types of IBDs, is different; however, it can include many therapy classes such as aminosalicylates, immunosuppressants (corticosteroids and cyclosporine), antimetabolites (i.e., azathioprine (AZA), 6-mercaptopurine (6 MP)), and biologic therapy (Eltantawy et al., 2023).

However, the evidence is changing rapidly; national and international guidelines are being updated continuously, and the pattern of biologic therapy use varies among different countries. Currently, the use of biologic therapies is recommended if conventional agents such as 5-aminosalisylic acids, corticosteroids, and immunomodulators fail (Gordon et al., 2024). Nonetheless, the initiation of biologic therapies in patients with IBD is mainly affected by disease severity, as well as other clinical factors. The increasing availability of biologic therapies makes it essential to understand the prevalence of their use, duration of therapy, and sequence of initiation to better optimize the treatment of IBD (Alulis et al., 2020).

The superiority of one biologic over another is unclear; there are few head-to-head clinical trials comparing the effectiveness of different biologic agents with each other, and given cost consideration and sample size, it is unlikely that many clinical trials will be performed in the near future (Laredo et al., 2022). The choice of biologic agents in biologic-naïve patients is primarily driven by patient preference, relative cost based on insurance coverage, and experience of the treating physician.

Real-world data assessing the effectiveness of biologics in biologic-naïve patients with IBD in the Middle East region are not well-established. Therefore, this study aims to assess the effectiveness of biologics (adalimumab, infliximab, ustekinumab, golimumab, and vedolizumab) for treating biologic-naïve patients with moderately to severely active IBD.

## Methods

### Patient or public involvement

No patient or public involvement.

### Study design and patient population

This study was a retrospective, observational study that involved chart reviews of patients with inflammatory bowel disease (IBD) at two tertiary care centers in Kuwait, Haya Alhabib Gastroenterology Center and Farwaniya Hospital. The enrollment period was between October 2017 and October 2023.

The study inclusion criteria were as follows: 1] age ≥ 18 years; 2] patients with moderate-to-severe ulcerative colitis defined as a clinical Mayo score of >6, with an endoscopic score of 2–3 (Feuerstein et al., 2020); 3] patients with moderate-to-severe Crohn’s disease defined as a Crohn’s disease activity index [CDAI] >220); with a simple endoscopic score for Crohn’s disease (SES-CD) ≥ 7 (Lichtenstein et al., 2018); 4] patients receiving adalimumab, infliximab, ustekinumab, or vedolizumab; 5] patient had been on the current biologic therapy between 6 weeks and 12 months of treatment, and 6] patient should not have received prior biologic therapy (biologic naïve).

Patients who did not continue their treatment for 12 months due to primary or secondary treatment failure were considered not to achieve endoscopic remission. In addition, if they were hospitalized, received corticosteroids, or had surgery due to medication failure before 12 months of therapy, they were considered not to have achieved the outcome and thus were counted as a failure.

Exclusion criteria included: 1] Patients who had been treated with biologic therapy previously (biologic experience); 2] patients with incomplete outcome or therapy data; 3] patients who received other concomitant biologic or small-molecule therapy for other conditions, for example, rheumatological disease, 4] pregnant patients, 5] patients who had intermittent suspension of therapy during the 12 month period.

### Outcomes and definitions

The primary endpoints were the percentage of hospitalization, surgery, corticosteroids-free remission, and endoscopic remission in patients with IBD receiving biologic therapies at week 52. Patients were considered to be on steroids if they received a course of prednisolone, budesonide, or any steroidal medication 6 weeks after starting the current biologic, that is, excluding the induction corticosteroid course. Patients who did not receive any steroid courses after 6 weeks from starting the biologic were considered to be in corticosteroid-free remission. Endoscopic remission is regarded as the total number of patients who achieved endoscopic remission, defined as an endoscopic Mayo score of 0–1 for patients with ulcerative colitis (Feuerstein et al., 2020) and a simple endoscopic score for Crohn’s disease (SES-CD) of 0–2 for Crohn’s disease (Lichtenstein et al., 2018). The number of patients with surgeries is the number of patients who underwent inflammatory bowel-related surgeries 6 weeks or more after starting the current biologic. Location and type of surgery were reported if patients had IBD-related surgery. Hospitalization, on the other hand, is the number of patients hospitalized 6 weeks or more after starting the current biologic for an IBD-related issue or complication. Examples of reasons for IBD-related issues include but are not limited to exacerbation of IBD, IBD-related infection, or any hospitalization, either due to IBD-related symptoms or complications.

This study was performed and reported in accordance with Strengthening the Reporting of Observational Studies in Epidemiology (STROBE) guidelines (von Elm et al., 2007). The international classification of diseases (ICD-10 version:2016) was used to make the diagnosis of IBD. Patients were considered to have IBD when they had ICD-10 K50, K50.1, K50.8, or K50.9 corresponding to Crohn’s disease (CD) and ICD-10 K51, K51.0, K51.2, K51.3, K51.5, K51.8, or K51.9 corresponding to ulcerative colitis (UC) (World Health Organization, 2022). The following baseline patient data were extracted from the clinical records and entered into a common database: sex, age, ethnicity, IBD type, body weight, duration of disease, smoking status, location, co-morbidities, and previous IBD medications.

### Ethical considerations

All methods were carried out in accordance with relevant guidelines and regulations. This study was reviewed and approved by the Ethical Review Board of the Ministry of Health of Kuwait (reference:3616, protocol number 3678/2021). All methods were carried out according to the guidelines and regulations of the Declaration of Helsinki and of the US Federal Policy for the Protection of Human Subjects. Patient consent was waived by the Ethical Review Board of the Ministry of Health of Kuwait.

### Statistical analysis

Statistical analyses were executed with the IBM SPSS Statistics package (Version 25.0. Armonk, NY: IBM Corp). Descriptive statistics were used to calculate frequencies and central tendency, expressed as means with standard deviation (SD), median with interquartile range (IQR), and percentages. Covariates included in the study were CRP, fecal calprotectin, and albumin because of their effect on disease activity (Turner et al., 2021).

## Results

**FIGURE 1:**
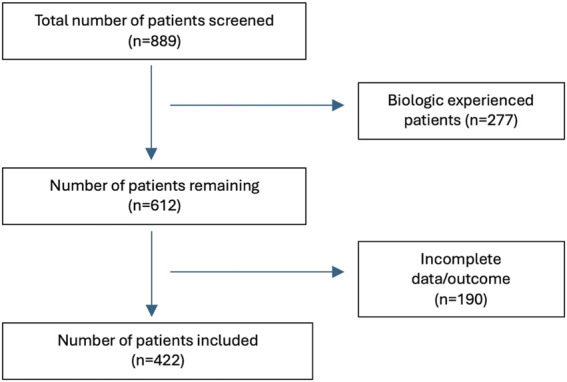
Flow diagram showing the enrollment process.

Initially, 889 patients on biologic therapies of interest were screened. Of this group, 277 were excluded because they were biologic experienced. Of the remaining 612 patients, 190 patients were excluded due to incomplete data. Of those 190 patients, 103 (54.2%) patients were excluded because they had not yet reached the week 52 timepoint (Figure 1). Therefore, a total of 422 patients were included in the study, of which 264 (62.5%) patients had Crohn’s disease (CD), and 158 (39%) had ulcerative colitis (UC). The median timepoint for endoscopic remission was 52 weeks ± 2.

In patients with CD, the mean age (SD) was 33.9 (10.2) years, and approximately half were male patients 136 (51.5%). Of the CD patients, 99 patients were on adalimumab, 72 were on infliximab, 61 were on ustekinumab, and 32 were on vedolizumab. The mean (SD) CRP (mg/L) and albumin (g/L) were 15.5 (6.3) and 40 (5.6), respectively. The mean stool fecal calprotectin (mcg/g) in patients with CD was 274 (14.5). Previous medications included azathioprine 47 (52.4%), methotrexate 22 (25.5%), and 6-mercaptopurines 20 (22.1). Co-morbidities in the CD cohort included diabetes (6.3%), hypertension (4.3%), and cardiovascular disease (6.0%). The demographic characteristics of patients with CD are described in Table 1.

**TABLE 1:** Demographic characteristics of patients with Crohn’s disease.

Crohn’s disease (n = 264)	Baseline	Follow-up
Age (years), mean (SD) At the time of study At diagnosis	33.9 (10.2) 32.1 (7.7)	— —
Sex, n (%) Male Female	136 (51.5%) 128 (48.5%)	— —
Ethnicity, n (%) Mediterranean Others	248 (94.0%) 16 (6.0%)	— —
BMI m^2^/kg, mean (SD)	24.9 (7.3)	—
CDAI, mean (SD) SES-CD, mean (SD)	318 (6.1) 11 (3)	181 (3.4) 1.7 (1)
L1: ileal L2: colonic L3: ileocolonic L4: upper gastrointestinal P: perianal B1: inflammatory B2: stricturing B3: penetrating	137 (52%) 26 (10%) 96 (36%) 5 (2%) 44 (16.8%) 124 (47%) 55 (21%) 85 (32%)	— — — — — — — —
Co-morbidities Diabetes Osteoarthritis Hypertension Cardiovascular disease Asthma	17 (6.3%) 13 (4.8%) 11 (4.3%) 16 (6.0%) 19 (7.2%)	— — — — —
Laboratory tests, mean (SD) CRP, mg/L Stool fecal calprotectin, mcg/g Albumin, g/L	15.5 (6.3) 274 (14.5) 40 (5.6)	9 (4.3) 16 (15.7) 40 (5.3)
Current biologics n (%) Adalimumab Infliximab Ustekinumab Vedolizumab Concomitant immunomodulator use	99 (37.5%) 72 (27.2%) 61 (32.1%) 32 (12.1%) 57 (21.5%)	— — — — —
Previous medications n (%) Immunomodulators Azathioprine Methotrexate 6-Mercaptopurines	89 (33.9%) 47 (52.4%) 22 (25.5%) 20 (22.1%)	— — — —

Crohn’s disease activity index [CDAI] with a simple endoscopic score for Crohn’s disease (SES-CD).

Of the patients with UC, 72 patients were on infliximab, 51 were on adalimumab, 21 were on vedolizumab, and 14 were on ustekinumab. In patients with UC, the mean age (SD) was 34.5 (11.4) years, and approximately half were male patients 136 (51.5%). Mean (SD) CRP (mg/L) and albumin (g/L) were 16.3 (5.2) and 42 (4.8), respectively. The mean stool fecal calprotectin (mcg/g) in patients with UC was 277 (11.6). Previous medications included 5-aminosalicylates 103 (65%) and immunomodulators 46 (29%). Co-morbidities in the UC cohort included osteoarthritis (6.3%), diabetes (7.7%), hypertension (5.9%), and asthma (8.0%). The demographic characteristics of patients with UC are described in Table 2.

**TABLE 2:** Demographic characteristics of patients with ulcerative colitis.

Ulcerative colitis (n = 158)	Baseline	Follow-up
Age (years), mean (SD) At the time of study At diagnosis	34.5 (11.4) 33.1 (8.7)	— —
Sex, n (%) Male Female	86 (54.3%) 72 (45.7%)	— —
Ethnicity, n (%) Mediterranean Others	145 (91.5%) 13 (8.5%)	— —
BMI m^2^/kg, mean (SD)	25.4 (6.9)	—
Mayo score, mean (SD) Mayo endoscopic score (MES), mean (SD)	8.8 (2.1) 2.5 (0.2)	3 (1.3) 1.3 (0.3)
E1: ulcerative proctitis E2: left-sided colitis E3: extensive colitis	30 (19%) 52 (34%) 76 (49%)	— — —
Co-morbidities Diabetes Osteoarthritis Hypertension Cardiovascular disease Asthma	12 (7.7%) 10 (6.3%) 9 (5.9%) 4 (2.6%) 13 (8.0%)	— — — — —
Laboratory tests, mean (SD) CRP, mg/L Stool fecal calprotectin, mcg/g Albumin, g/L	16.3 (5.2) 277 (11.6) 42 (4.8)	9.5 (4.1) 16 (12.5) 40 (4.1)
Current biologics n (%) Adalimumab Infliximab Ustekinumab Vedolizumab Concomitant immunomodulator use	51 (32.2%) 72 (45.5%) 14 (8.8%) 21 (13.2%) 58 (36%)	— — — — —
Previous medications n (%) 5-Aminosalicylates Immunomodulators	103 (65%) 46 (29%)	— —

### Crohn’s disease outcomes

In patients with CD, steroid-free remission was achieved in 65 (66%) of the patients on adalimumab, 50 (69%) on infliximab, 41 (68%) on ustekinumab, and 21 (65%) on vedolizumab. Additionally, endoscopic remission was attained in 51 (52%) of the patients on adalimumab, 38 (53%) of the patients on infliximab, 34 (56%) of the patients on ustekinumab, and 16 (51%) patients on vedolizumab. Some patients experienced primary (15 patients) and secondary (33 patients) non-response while taking adalimumab. Ten patients experienced primary non-response while taking infliximab, and 24 patients experienced secondary non-response. Seven patients taking ustekinumab experienced primary non-response, and 20 patients experienced secondary non-response. Five patients taking vedolizumab experienced primary non-response, and 11 patients experienced secondary non-response.

**FIGURE 2:**
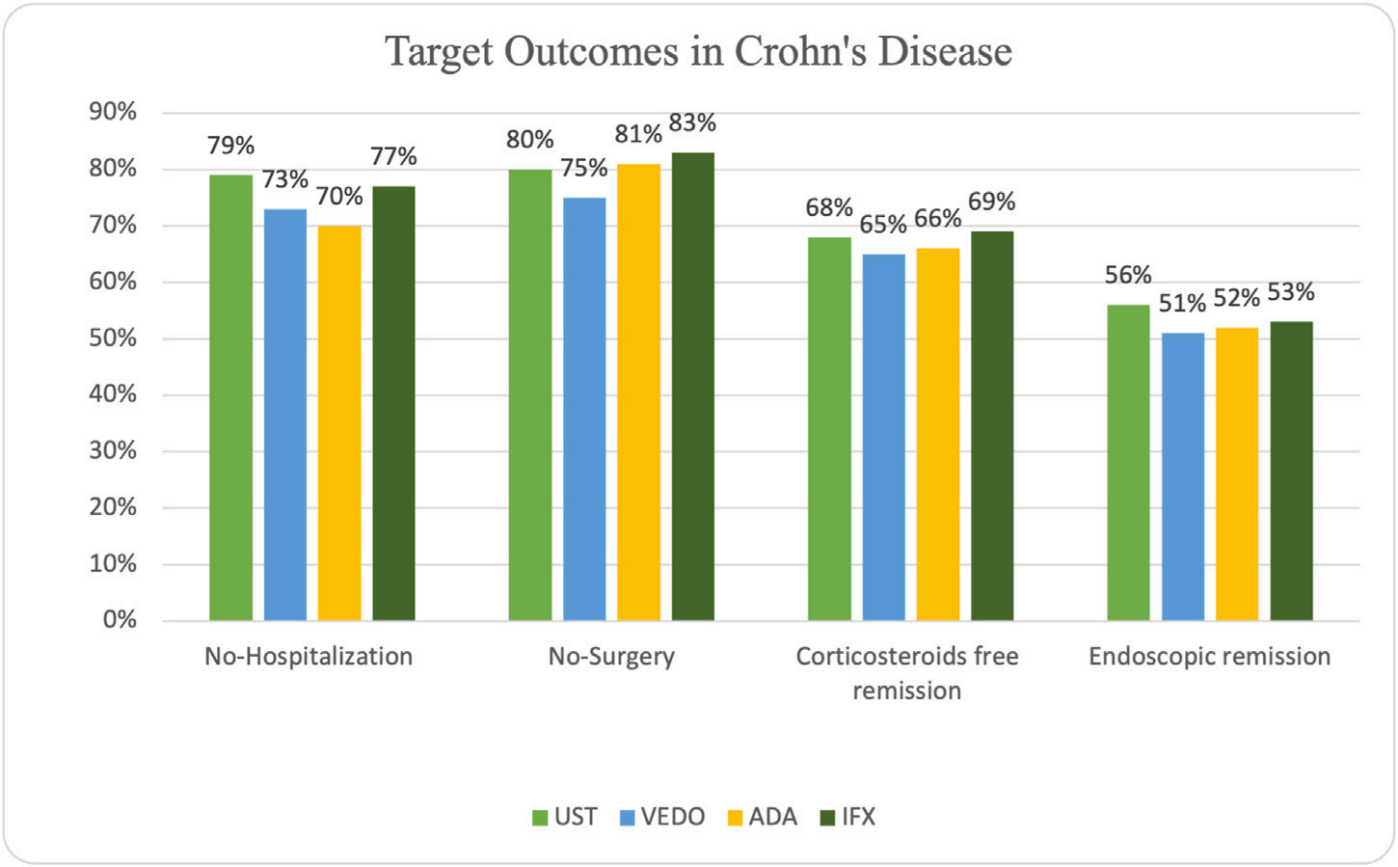
Graph depicting outcomes in biologic-naïve patients with Crohn’s disease.

IBD-related hospitalization occurred in 30 (30%) of the patients on adalimumab, 17 (23%) patients on infliximab, 13 (21%) patients on ustekinumab, and 9 (27%) patients on vedolizumab. IBD-related surgery occurred in 19 (19%) patients receiving adalimumab, 12 (17%) patients on infliximab, 12 (20%) patients on ustekinumab, and 8 (25%) patients receiving vedolizumab (Figure 2).

In total, 51 of the 264 patients (19.3%) underwent surgery (small bowel resection ± right hemicolectomy, see Table 3).

**TABLE 3:** Patients with Crohn’s disease who had IBD-related surgery.

	Small bowel resection	Small bowel resection + right hemicolectomy
Adalimumab (n = 19)	14	5
Infliximab (n = 12)	9	3
Ustekinumab (n = 12)	10	2
Vedolizumab (n = 8)	5	3

### Ulcerative colitis outcomes

In patients with UC, steroid-free remission was achieved in 32 (62%) of the patients on adalimumab, 47 (65%) on infliximab, 9 (64%) on ustekinumab, and 13 (64%) on vedolizumab. Additionally, endoscopic remission was attained in 40 (56%) of the patients on infliximab, 26 (61%) of the patients on adalimumab, 8 (55%) of the patients on ustekinumab, and 11 (53%) patients on vedolizumab. Ten patients experienced primary non-response while taking infliximab, and 22 patients experienced secondary non-response. Six patients experienced primary non-response while taking adalimumab, and 19 patients experienced secondary non-response. One patient taking ustekinumab experienced primary non-response, and five patients experienced secondary non-response. Finally, four patients experienced primary non-response while taking vedolizumab, and six patients experienced secondary non-response.

**FIGURE 3:**
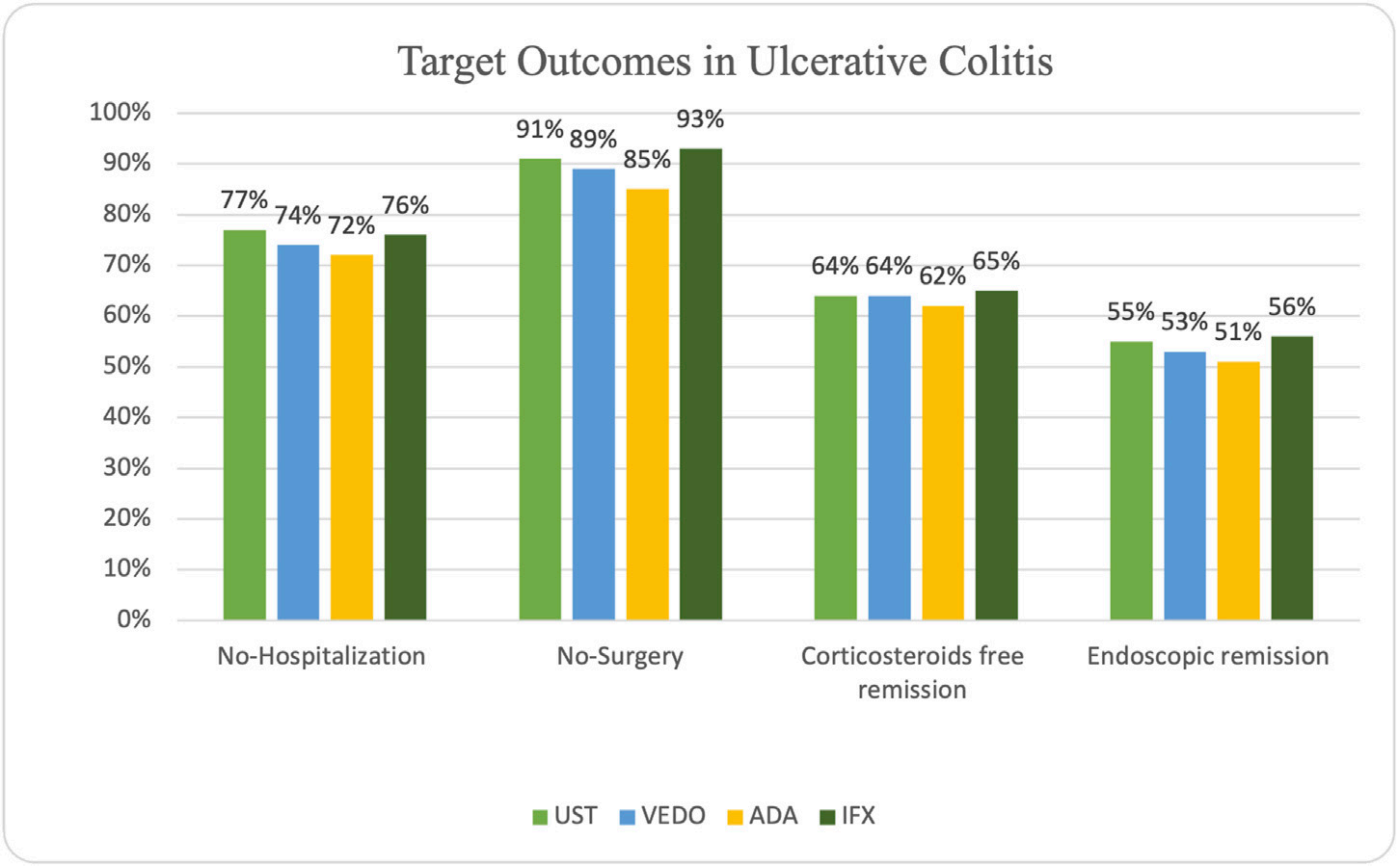
Graph depicting outcomes in biologic-naïve patients with ulcerative colitis.

IBD-related hospitalization occurred in 14 (28%) of the patients on adalimumab, 17 (24%) patients on infliximab, three patients on ustekinumab (23%), and 5 (26%) patients on vedolizumab. IBD-related surgery occurred in 8 (15%) patients receiving adalimumab, 5 (7%) patients on infliximab, 1 (9%) patient on ustekinumab, and 2 (11%) patients receiving vedolizumab (Figure 3).

In total, 17 of the 158 patients (10.8%) underwent surgery (colectomy followed by ileal pouch-anal anastomosis (IPAA) or proctocolectomy with end ileostomy, see Table 4).

**TABLE 4:** Patients with ulcerative colitis who had IBD-related surgery.

	Proctocolectomy with end ileostomy	Colectomy followed by IPAA
Adalimumab (n = 8)	1	7
Infliximab (n = 5)	0	5
Ustekinumab (n = 1)	0	1
Vedolizumab (n = 2)	1	1

IPAA, ileal pouch-anal anastomosis.

## Discussion

This study evaluated the effectiveness of biologic therapies in bio-naive patients with IBD. The primary outcomes were the percentage of hospitalization, surgery, steroid-free remission, and endoscopic remission, defined as a Mayo score of 1 or less in ulcerative colitis and an SES-CD score of less than 3 in Crohn’s disease. All biologic therapies were effective in achieving clinical and endoscopic clinical outcomes in IBD.

Our finding is similar to a study performed in the United Kingdom (Kapizioni et al., 2024). The study presented data on the real-world use of biologic therapy in 13,222 patients. The authors found that the effectiveness of adalimumab, infliximab, ustekinumab, and vedolizumab were similar in IBD.

In our study, the rate of endoscopic remission in biologic-naïve patients with CD receiving infliximab was 53%, whereas the rate of endoscopic remission in patients with UC receiving infliximab was 56%. One real-world study investigated similar outcomes in patients with CD receiving infliximab for 12 months, and the authors found that the long-term response rate was approximately 60% (Kestens et al., 2013).

In our study, endoscopic remission in patients receiving adalimumab was 52% in CD and 51% in UC. One study included 263 patients with UC (87 naïve and 176 previously exposed to anti-TNF). Similar to our study, after 12 weeks, the authors found that endoscopic remission in the naïve group was 50% (Iborra et al., 2017). In a Spanish cohort study of patients with UC, adalimumab therapy was associated with a clinical response rate of 61% in anti-TNF-naïve and 47% in anti-TNF-experienced patients (Iborra et al., 2017).

The present study showed that in patients receiving vedolizumab, 51% of the CD cohort and 53% of the UC cohort achieved endoscopic remission. One multicenter study demonstrated the effectiveness of vedolizumab as a first-line biologic in IBD in a real-world setting (Kopylov et al., 2018). The study reported that at week 14, 82% of CD and 79.1% of UC anti-TNF-naïve patients responded to treatment with vedolizumab. At the last follow-up, 77.1% of CD and 76.7% of UC patients responded to vedolizumab.

Several real-world studies (Kestens et al., 2013; Osterman et al., 2014; Cosnes et al., 2016; Bohm et al., 2020) concluded that infliximab and adalimumab appeared to have similar effectiveness in patients with CD, and the approval of vedolizumab and ustekinumab for CD expanded the options of biologics for moderate-to-severe disease. Two studies compare the safety and effectiveness of vedolizumab and TNF-antagonist therapy in adult patients with CD. Both studies indicated no significant difference in achieving disease remission (Bohm et al., 2020; Macaluso et al., 2021).

In our study, the proportion of biologic-naïve patients with UC who achieved corticosteroid-free remission after receiving infliximab or vedolizumab was 65% and 64%, respectively. A *post hoc* analysis of three UC clinical trial programs that included data on 795 biologic-naïve UC patients compared the efficacy of infliximab and vedolizumab for moderate-to-severe biologic-naïve UC (Narula et al., 2022). Differences in the proportions of patients achieving one-year corticosteroid-free clinical remission and endoscopic remission were reported. Rates of corticosteroid-free clinical remission were significantly higher in patients using infliximab (29.5%) than vedolizumab (15.0%, p= .004). Rates of 1-year endoscopic remission also were significantly higher in infliximab-treated patients (36.0% vs. 25.6% OR, 1.55; 95% CI, 1.08–2.22).

In terms of IBD-related surgery, our study showed that in patients with CD, 25% receiving vedolizumab and 20% receiving ustekinumab had undergone CD-related surgery. One study aimed to investigate the incidence of the first CD-related surgery following the initiation of treatment with vedolizumab or ustekinumab in biologic-naïve patients with CD (Onali et al., 2022). After 1 year of follow-up, the study reported that 7.7% of patients receiving vedolizumab and 11.6% of patients receiving ustekinumab had undergone a CD-related surgery. In patients with UC, the present study found that the proportion of patients who had surgery was 7% in patients receiving infliximab and 15% in patients receiving adalimumab. A nationwide study from Denmark compared the effectiveness of infliximab and adalimumab in biologic-naïve patients with UC. The study reported that the rate of abdominal surgery was 11 per 100 person-years in the infliximab cohort and 20 per 100 person-years in the adalimumab group (Singh et al., 2017).

One systematic review and network meta-analysis investigated the efficacy of different biologic therapies in patients with moderate-to-severe UC as a first-line choice. The meta-analysis included 12 RCTs, and they found that among biologic-naïve patients, infliximab and vedolizumab were ranked highest for induction of clinical remission and mucosal healing (Singh et al., 2018).

This study has several clinical implications. The widespread availability of different biologic therapies for patients with IBD adds challenges to the management of these patients. Currently, guidelines recommend either vedolizumab or anti-TNF therapy as first-line biologics in moderate-to-severe UC (Feuerstein et al., 2020; Rubin et al., 2019). Although the VARISTY trial (Sands et al., 2019) showed the superiority of vedolizumab compared to adalimumab in achieving clinical remission and endoscopic improvement in biologic-naïve patients with UC, it is still debated whether this superiority would hold against other anti-TNF therapies such as infliximab. Real-world data such as the present study help clinicians understand the effectiveness of biologics in achieving important clinical outcomes in patients with Arab ethnicity. Data from head-to-head trials would be ideal to understand and ascertain the effectiveness of biological therapies compared to each other and will aid in the generalization to different populations.

This study is not without limitations. First, it is a retrospective observational study; thus, generalization is not possible, and unmeasured confounding factors may be present. Second, we could not investigate the impact of dose escalation or therapeutic drug monitoring practices, which are common in practice. Third, a comparison between outcomes of different biologic agents was not assessed because the number of included patients was insufficient to perform such a comparison. Finally, a long-term evaluation of outcomes of more than 12 months was not assessed.

## Conclusion

Adalimumab, infliximab, ustekinumab, and vedolizumab were all effective in achieving clinical and endoscopic clinical outcomes in IBD in both UC and CD. The findings of this study suggest that the efficacy of biologics in the Middle East is similar to that in the Western population. However, larger prospective comparative studies are warranted.

## Data Availability

The original contributions presented in the study are included in the article/Supplementary Material; further inquiries can be directed to the corresponding author.
